# Ultrasonography Causes Agitation and Pain Leading to Hemodynamic Disturbance in Neonates: A Prospective Observational Study

**DOI:** 10.3390/children10020347

**Published:** 2023-02-10

**Authors:** Emre Dincer, Hamza Özer, Sevilay Topçuoğlu, Güner Karatekin

**Affiliations:** 1 Department of Neonatology, University of Health Sciences, Zeynep Kamil Maternity and Children’s Research and Training Hospital, Op. Dr. Burhanettin Öncel Cad No. 10, Üsküdar, Istanbul 34668, Turkey; 2 Department of Radiology, Bolu Abant İzzet Baysal University Medical Faculty, Bolu 14030, Turkey

**Keywords:** ultrasonography, pain, hemodynamics, near-infrared spectroscopy, Doppler ultrasonography

## Abstract

Background: Ultrasonography is widely used in neonatological practice and studies investigating the hemodynamic effects of various treatment protocols or clinical situations. On the other hand, pain causes changes in the cardiovascular system; so, in the case of ultrasonography leading to pain in neonates, it may cause hemodynamic alterations. In this prospective study, we evaluate whether ultrasonographic application causes pain and changes in the hemodynamic system. Methods: Newborns undergoing ultrasonographic examination were enrolled in the study. Vital signs, cerebral and mesenteric tissue oxygenation (StO_2_) levels, and middle cerebral artery (MCA) Doppler measurements were recorded, and NPASS scores were calculated before and after ultrasonography. Results: We enrolled 39 patients in the study. After ultrasonography, Neonatal Pain, Agitation, and Sedation Scale (NPASS) scores were significantly higher (*p* < 0.01), and all vital signs (heart rate, respiratory rate, SpO_2_, diastolic and systolic blood pressure; *p* = 0.03; *p* < 0.01, *p* < 0.01, *p* < 0.01, *p* = 0.02, *p* = 0.03, respectively) were altered. Cerebral (*p* = 0.008) and mesenteric (*p* = 0.039) StO_2_ levels were significantly lower in the whole study group, MCA end-diastolic velocity decreased (*p* = 0.02), and the resistive index (*p* = 0.03) increased in patients whose NPASS score was >7 after ultrasonography. Conclusions: This study is the first to show that ultrasonography may cause pain in newborn patients, and alters vital signs and hemodynamic parameters. Therefore, precautions should be taken to protect newborn babies from pain during ultrasound applications, as they are already exposed to many noxious stimuli. Furthermore, pain scores should be considered in studies using ultrasonography and evaluating hemodynamic parameters to increase the reliability of the studies.

## 1. Introduction

In recent years, ultrasonography (USG) has increased its popularity in neonatological practice [1]. Cranial USG and targeted echocardiography have been widely used in clinical practice [1,2]. Lung USG can be used to diagnose lung pathologies in term and preterm babies. On the other hand, recent studies showed that intestinal USG can guide clinicians in patient follow-up and treatment planning, especially in necrotizing enterocolitis [3]. In addition, USG is effective in confirming the location of umbilical catheters, peripherally inserted central catheters, and intubation tubes. Therefore, ultrasound is now considered a “second stethoscope”, and its clinical impact is expected to increase in the following years [2].

Another benefit of USG is providing information about visceral blood flow with Doppler studies. Doppler USG is frequently used to assess cerebral, renal, or mesenteric blood flow in clinical conditions such as patent ductus arteriosus and hypoxic–ischemic encephalopathy. Doppler USG is also used to evaluate the hemodynamic effects of drug applications or treatment protocols [4].

However, a study conducted in 2017 showed that echocardiography causes pain in newborn babies and changes in vital signs [5]. In 2022, Ahsan et al. also reported that targeted neonatal echocardiography causes significant pain or discomfort and physiological instability in neonates [6]. In these studies, pain scores were significantly higher, SpO_2_ levels decreased, and the heart rate increased significantly after echocardiography [5,6]. In another study, oral sucrose reduced pain caused by echocardiographic applications [7].

Acute and chronic pain is an important but underestimated issue in NICU [8]. An infant may be exposed to more than 20 procedures causing acute pain [9,10], and more than 1000 h of cumulative chronic stressors in long-term hospitalization [10]. Although the procedures are not always harmful as the heel-stick test, catheter placement, or intubation is, interventions such as diaper changes, positioning the infant, or echocardiographic applications [5] may also cause stress and stress responses [11]. The more newborns are exposed to these acute stimuli and the longer they are exposed to chronic stressors, the more likely they are to have neurodevelopmental problems [12]. 

Exposure to stress or pain activates the sympathetic autonomic nervous system and prepares the body to a “fight or flight” response with the hypothalamic–pituitary–adrenal axis. The “fight” response is generated by the sympathetic nervous system by the production of adrenaline, and the “flight” response is generated by the hypothalamic–pituitary–adrenal axis with the secretion of steroidal stress hormones, primarily cortisol. However, newborn infants cannot produce these responses adequately—they can neither fight nor fly—and repetitive pain and chronic stress may cause a diversion of energy from essential brain development [11]. Painful procedures decrease brain function as a result of activating a downstream cascade of stress signaling, and their numbers are negatively correlated with head growth [13]. 

Alongside the effects on neurological development, studies examining hemodynamic responses to pain have shown that acute pain causes alterations in heart rate, systolic and diastolic blood pressure, and even oxygen saturation values [14]. Increments of catecholamine levels with acute pain were also shown in newborn studies, which may lead to changes in the hemodynamic system [15].

Neonatal Pain, Agitation, and Sedation Scale Scoring (NPASS), which is recommended to be used in the evaluation of pain and sedation in newborns, has a criterion for a change in vital signs [16]. NPASS is a pain scoring system recommended by the American Academy of Pediatrics that demonstrated good reliability and validity in systematic reviews. It can be used in different neonatal subpopulations, including all gestational ages and mechanically ventilated or nonventilated infants [17]. 

While USG and Doppler studies are used to investigate the hemodynamic effects of any factor, they have the potential to cause hemodynamic changes if they cause pain in a neonate. Therefore, if ultrasonographic application is proven to cause pain or agitation in this group of patients, it is also logical to consider administering analgesic agents. 

In light of this knowledge, our study primarily aimed to investigate whether USG applications cause a pain response and changes in vital signs in newborn babies. In addition, we aimed to investigate whether USG application causes changes in middle cerebral artery flow, and cerebral and mesenteric tissue saturation values measured with near-infrared spectroscopy (NIRS).

## 2. Materials and Methods

This prospective, observational, self-controlled clinical study was conducted between January 2021 and February 2022 in a tertiary hospital’s neonatal intensive care unit with 1500 annual admissions. The institutional ethics committee approved the study, and parental consent was obtained for all the patients.

### 2.1. Patient Enrollment

All patients who had undergone consecutive abdominal and transfontanelle USG in the same session during the study period were included. Clinically stable infants with no hemodynamic problems (such as hospitalized patients due to the transient tachypnea of the newborn and recovered in a short time, or who were hospitalized for hyperbilirubinemia or social reasons) were chosen for enrollment. 

Newborn patients with transient tachypnea who have had mechanical ventilation are routinely screened with transcranial ultrasonography. In these patients, antenatal features such as intestinal echogenicity increment and Grade I hydronephrosis underwent abdominal ultrasonography. In indirect hyperbilirubinemia, patients with no risk factors for jaundice are routinely screened with cranial and abdominal ultrasonography for hemorrhage. Abandoned babies to be given to social services are routinely screened with ultrasonography. Patients who had undergone ultrasonography for these indications were included in the study.

Patients under sedative medication, who had invasive or noninvasive mechanical ventilation support, cardiovascular instability, needed inotropic medication, had Grade II–III intraventricular hemorrhage or periventricular hemorrhagic infarction, major congenital anomalies, and congenital heart diseases were excluded from the study. Patients were evaluated after the fourth day of life to eliminate the effects of the transitional period and patent ductus arteriosus on neonatal hemodynamics. Patients who were too small or large for their gestational age and with fetal growth restrictions were also excluded from the study.

### 2.2. Pain Evaluation

The NPASS was used to evaluate the patients’ pain as recommended in our national neonatology society guidelines [16]. 

The NPASS consists of five categories: crying/instability, behavioral state (spontaneous movements, kicking or arching), facial expression, extremities tone, and vital signs. In each category, the observer scores the patient between −2 and 2. All scores are summed up, and the patient is scored between 10 and −10. Values below zero indicate a sedated patient, while positive and high values indicate a patient with agitation and pain. In general, scores between 1 and 3 are considered mild pain, 4–6 are moderate, and scores >6 are considered severe pain [18]. 

E.D. recorded the babies and patient monitors on video with a smartphone before and after USG. To avoid intraobserver variability, two researchers, G.K. and S.T., calculated NPASS scores blinded from Doppler USG and NIRS values. In addition, G.K. and S.T. calculated the NPASS scores one week after the first calculation to avoid intraobserver variability. 

### 2.3. Ultrasonography

The same radiologist (H.Ö.) performed all USG applications and Doppler evaluations while blinded from NPASS scores, vital signs, and StO_2_ levels recordings. Hand hygiene was ensured before beginning the USG procedure for all patients. 

In our clinic, ultrasound gels were filled in 10 cc injectors and stored in the patient’s incubator to keep the gels constantly warm and ensure infection control by using personalized gels. In this way, radiologists and clinicians used each infant’s own gel for ultrasound applications. 

Ultrasonographic examinations and Doppler measurements were conducted with a pulse-wave Doppler ultrasound (Philips Affinity 50, Philips, Bothell, WA, USA) with a 5–12 MHz pediatric sector transducer. 

Blood flow velocities were measured in the left or right middle cerebral artery (MCA). The measurements were performed through the anterolateral fontanelle. The probe was placed above the ear, 1 cm anterior and superior to the external auditory meatus, to find the temporal window. The orbit and the zygomatic arch were used for the horizontal position marker. The angle between the blood flow direction and the ultrasound beam was kept below 15° [19]. MCA peak systolic velocity (PSV) and end-diastolic velocity (EDV) were measured (cm/s) for all neonates included in the study with a pulse-wave Doppler ultrasound (Philips Affinity 50, USA) with a 5–12 MHz pediatric sector transducer over the temporal window of the right MCA. 

After the first Doppler measurement, routine cranial and abdominal control ultrasound examinations were performed. The second Doppler measurements were then performed. The resistive index was calculated using the formula of (PSV − EDV)/PSV [19]. 

No analgesic treatment (pharmacologic or nonpharmacologic) was applied during the examinations, but optimal handling precautions were taken. The babies could not be swaddled or cuddled to not prevent abdominal ultrasonography. To minimize agitation and pain, all the babies were given pacifiers, and the incubators were not opened, so that the incubator temperature would not decrease. The application was made by opening one of the small incubator windows. 

### 2.4. Vital Sign Recordings

The patients’ heart rate, respiratory rate, SpO_2_, and systolic–diastolic blood pressure were recorded just before USG application and as soon as the USG had been completed. A multiparameter patient monitor (OKM800, OKUMAN, Ankara, Turkey) was used for vital sign monitoring. 

### 2.5. NIRS Evaluation

Near-infrared spectroscopy recorded cerebral (StcO_2_) and mesenteric (StmO_2_) tissue oxygenation through an INVOS™ Oximeter (Somanetics, Covidien, Mansfield, MA, USA). Cutaneous sensors were applied on the forehead for StcO_2_ and the infraumbilical area for StmO_2_ 1 h before the first evaluation. Stable StcO_2_ and StmO_2_ values were recorded just before USG (*before*) and as soon as USG was finished (*after*). Fractional oxygen extraction (FOE) was calculated with the formula (SpO_2_ − StO_2_)/SpO_2_ [20].

### 2.6. Timeline

Ultrasonographic evaluations are planned during morning patient rounds in our clinic, and the radiology department is informed. One hour before the USG, NIRS probes were placed. When the radiologist was ready for USG, E.D. recorded the patient’s vital signs and NIRS values, and recorded the baby on the video with a smartphone to allow for G.K. and S.T. to calculate the NPASS score. Then, the radiologist produced the MCA Doppler USG, transfontanelle USG, abdominal USG, and again the MCA Doppler USG in the same order for all patients. As soon as the USG procedure had finished, vital signs and StO_2_ levels were recorded, and the NPASS score was calculated again as before the USG.

### 2.7. Statistical Analyses

For statistical analyses, SPSS software version 20.0 was used. The variables were investigated using visual (histograms, probability plots) and analytical (Shapiro–Wilk, Kolmogorov, and Smirnov tests) methods to determine whether they were normally distributed. Parametric results are expressed as mean ± standard deviations. The median values of nonparametric tests are reported with median and minimal–maximal values. Non-normally distributed dependent variables were compared with the Wilcoxon test, and the paired *t*-test was performed for dependent parametric variables. 

## 3. Results

During a study period of 13 months, only one patient per week could be included in the study because of the radiologist’s availability. As a result of this, 44 patients were included in the study. However, two patients were excluded because of Grade II intraventricular hemorrhage, and two were excluded because of congenital heart disease (one hemodynamically significant patent ductus arteriosus and one ventricular septal defect). In addition, one of the patients was excluded because of clinical deterioration with apnea and the need for bag-mask ventilation. We included 39 successful evaluations in the study. Of the patients, 56% (n: 22) were male. The median gestational age was 37 weeks (min: 28, max: 41; 25th–75th percentiles: 34–38 weeks), and the median birth weight was 3000 g (min: 1280, max: 2880; 25th–75th percentiles: 2360–3340 g). The median postmenstrual age was 38 (min: 31, max: 41; 25th–75th percentiles: 35–39) weeks. The median chronological age was 6 (min: 4, max 27; 25th–75th percentiles: 4–9) days (Table 1). 

All vital signs were significantly altered after the USG application. Heart rate (134.1 ± 12.6 to 143.3 ± 14.8; *p* < 0.01), respiratory rate 40.4 ± 5.9 to 46.6 ± 7.3; *p* < 0.02), and systolic blood pressure (68 ± 5.6 to 71.3 ± 6.3; *p* = 0.02) and diastolic blood pressure (median 36, IQR: 9 to median: 41 IQR: 9; *p* = 0.03) significantly increased after USG, while SpO_2_ significantly decreased (median: 100 IQR: 2 to median: 96 IQR: 6; *p* < 0.01).

The pain scores of the patients were significantly higher after USG than those before USG (before: median: 1 IQR: 1; after: median: 5 IQR: 5; *p* < 0.01. Vital signs and NPASS scores comparisons are depicted in Table 2).

Cerebral and mesenteric tissue saturation values measured after the USG were significantly lower than those before the application (before: 98 ± 10; after: 74 ± 12; *p* = 0.08. Before: 75 ± 32; after: 70 ± 32; *p* = 0.039) (Table 3). 

There was no significant difference in Doppler measurements of the patients regarding PSV, EDV, and RI (*p* = 0.807, *p* = 0.143, and *p* = 0.065, respectively) (Table 4).

On the other hand, in patients whose NPASS score was ≤4 before and increased above 4 after USG, EDV was significantly decreased after USG (*p* = 0.022). In addition, in patients whose NPASS score was ≤7 and increased above 7 after USG, both EDV and RI were significantly decreased after USG (*p* = 0.025, *p* = 0.036) (Table 5).

The mean duration of USG applications was 660 ± 111 s. There was no correlation between USG duration and pain scores observed after USG (Pearson correlation: 0.143, *p*: 0.384).

## 4. Discussion

Our study shows that USG application causes an increase in the pain scores, and changes in the vital signs and StO_2_ levels of newborn babies. Additionally, we observed that the MCA flow pattern changed in infants whose pain scores significantly increased. 

In studies evaluating the hemodynamic results of various applications in newborns, vital signs [21], NIRS values [22], and Doppler USG measurements [4] are frequently used. Studies evaluating the hemodynamic effects of drug administrations, treatment protocols, or previously diagnosed pathologies such as fetal growth restriction, patent ductus arteriosus, or anemia are often combined with ultrasonography and Doppler measurements [23]. However, if the USG application changes these parameters, it may raise questions about the results of these evaluations. 

Awareness of newborns feeling pain has greatly increased in recent years: while it was thought until the mid-1900s that newborns did not feel pain, it has been proven in recent years that pain causes disorders in neurodevelopmental outcomes. Various pain scoring systems are used in NICUs, but NPASS is the only pain scale designed for measuring acute and prolonged pain, and considers sedation [21]. 

Animal studies have shown that recurrent or persistent pain causes apoptosis in neurons. Furthermore, studies have shown that the allostatic loads of premature babies constantly exposed to pain and stress in the newborn period, having immature physiological and stress systems, increase significantly, and this situation causes neurodevelopmental problems in the long term. In studies in the neonatal period, increased catecholamine, growth hormone, glucagon, cortisol, and aldosterone levels, and the suppression of insulin secretion were found in acute pain [16,24].

In the short term, pain stimulates the sympathetic arm of the autonomic nervous system, which results in increased heart rate, blood pressure, respiratory rate, and intracranial pressure, decreased oxygen saturation, and the sweating of the palms. In addition to these changes, there are changes in the respiratory pattern, skin color, and pupil size following painful stimuli. In our study, pain scores were significantly higher after USG application than those before, and increased pain or stress may have led to these changes in the babies’ vital signs [14,16].

In previous studies, cumulative pain and stress were correlated with the risk of neurodevelopmental problems [10]. In these studies, all invasive procedures such as intubation, blood sampling, catheter placement, and invasive and noninvasive mechanical ventilation applications, and harmless interventions such as positioning the infants and diaper changes [10,21] or adhesive removal [9] were considered acute/chronic pain sources or stress causes [10]. As a result of our study and previous echocardiographic studies [4,5,6], ultrasonographic and echocardiographic applications should also be accounted for as pain/stress causes in future studies besides hemodynamic effects.

Previous studies investigating NIRS and pain reported different results. Studies using NIRS have demonstrated that oxygenated and total hemoglobin increased in the contralateral hemisphere after noxious stimuli, indicating functional cerebral hyperemia. In the study of Dix et al., a “bilateral” cerebral hemodynamic response following painful stimuli was reported. It is generally concluded that cerebral tissue oxygenation and cerebral blood flow increase due to cortical activation, secondary to pain [25]. On the other hand, in a study by Hwang et al., cerebral tissue oxygenation decreased after the heel-stick procedure [26]. In another study, cerebral StO_2_ values increased on the contralateral side after the heel-stick procedure; however, interestingly, in the same study, StO_2_ values decreased on the ipsilateral side, but this was not discussed separately in the article [27]. 

In our study, both cerebral and mesenteric StO_2_ levels decreased significantly after the USG procedure, but there was no significant change in FOE values. In light of previous studies, it was expected that StO_2_ [25] and FOE values would increase [26], but SpO_2_ levels would decrease [5] after noxious stimuli. However, considering that the FOE formula was (SpO_2_ − StO_2_)/SpO_2_, our patients’ FOE levels did not change, but there was a significant decrease in SpO_2_ levels. Therefore, we assume that oxygen extraction did not change after ultrasonographic application, but SpO_2_ decreased in our patients, which led to a decrease in StO_2_ levels. Nevertheless, the pathophysiology of StO_2_ and FOE responses to pain and noxious stimuli must be studied in further studies investigating oxygen delivery, consumption, and cerebral vascular resistance.

On the other hand, in light of the current literature, no study has evaluated StO_2_ values rather than cerebral StO_2_, and this is the first study to evaluate mesenteric StO_2_ levels after ultrasonography. 

In our study, when infants were grouped according to NPASS scores after USG application, in the group of patients whose pain scores increased significantly (NPASS scores > 7 after the procedure, which is considered severe pain or agitation [18]), EDV values decreased, and RI increased significantly. This group of patients could also be considered to have had a more intense response to pain or stress stimuli. In this regard, we can speculate that, in these patients, the sympathetic nervous system is activated due to pain and agitation or unwanted stimuli, and increased catecholamine levels may have led to vasoconstriction, decreased EDV values, and increased RI [24,28]. However, further studies are also needed to explain cerebral hemodynamic responses to pain, as mentioned before.

Our study has several limitations. Studies with a larger number of patients may better evaluate if there is a significant change in PSV and FOE. Our study group was heterogeneous regarding gestational week and chronological age. A more homogeneous study group may provide better results because the response to pain and discomfort is different between infants of different gestational ages [14,16] and exposed to repeated painful procedures [27]. On the other hand, we did not investigate how long the hemodynamic changes and increased pain scores lasted after the application. Future studies evaluating these pain and stress responses to USG application without these limitations may further clarify this relationship. However, this study is the first to demonstrate that USG applications lead to changes in vital signs, tissue oxygenation, and cerebral Doppler findings. 

In fact, NPASS, or other pain scoring systems or scales do not distinguish pain from agitation and discomfort. Therefore, in our study, the results may have not only been due to pain, but also due to the discomfort or agitation caused by the application [29]. On the other hand, whatever the reason may be, this situation caused by ultrasonography causes changes in the parameters in the patient.

In conclusion, ultrasonographic application may cause pain, agitation, or discomfort in newborn patients, leading to alterations in vital signs, NIRS values, and MCA Doppler studies. Therefore, during ultrasound applications, precautions should be taken to protect newborn infants who are already exposed to many noxious stimuli in the NICU from pain. First, by forming a clinical or even national protocol on ultrasound indications, unnecessary ultrasound applications can be avoided, and the total number of ultrasound applications can be reduced. Although point-of-care ultrasonography has recently been encouraged and shown to be helpful in many scenarios in NICU [30], clinicians should keep in mind that ultrasonography may cause pain or stress in infants. In patients undergoing USG, nonpharmacological methods such as swaddling, kangaroo care, and breastfeeding should be considered during application [31]. If these precautions are not feasible, analgesic agents such as oral sucrose may be administered to reduce pain [32]. Furthermore, while using USG and evaluating hemodynamic parameters, it is important to consider pain scores to increase the reliability of the measurements in daily clinical practice.

## Figures and Tables

**Table 1:** Demographic data of the patients (values not normally distributed are given with the median, and the 25th and 75th percentiles, and marked with *).

n: 39	Mean	±SD (25th–75th%)
Gestational age * (weeks)	37	34–38
Birth weight * (gr)	3000	2360–3340
Postnatal age * (days)	6	4–9
Postmenstrual age * (weeks)	38	35–39
Actual weight	2841	659
Sex: female/male	17/22	

**Table 2:** The patients’ vital signs and pain scores before and after ultrasonographic application (not normally distributed values are given with the median, and 25th and 75th percentiles, and marked with *).

Variable	Mean (before)	±SD	Mean (after)	±SD	*p*
Heart Rate (/min)	134.1	12.6	143.3	14.8	<0.01
Respiratory rate (/min)	40.4	5.9	46.6	7.3	<0.01
SpO_2_ (%) *	100	(98–100)	96	(93–99)	<0.01
Systolic blood pressure (mmHg)	68.0	5.6	71.3	6.3	0.02
Diastolic blood Pressure * (mmHg)	36	(35–44)	41	(38–47)	0.03
NPASS score *	1	1	5	5	<0.01

**Table 3:** NIRS measurements, calculated FOE of the patients. Abbreviations: StO_2_: tissue oxygen saturation, FOE: fractional oxygen extraction.

	Before USG Application		After USG Application		
Variable	Median	25–75th%	Median	25–75th%	*p*
Cerebral StO_2_	98	75–91	74	71–83	0.008
Cerebral FOE	19	14–26	20	15–25	0.185
Mesenteric StO_2_	75	53–85	70	53–85	0.039
Mesenteric FOE	28	14–44	29	15–46	0.558

**Table 4:** Doppler measurements of the patients before and after ultrasound application. Abbreviations: PSV: peak systolic velocity, EDV: end-diastolic velocity, RI: resistive index.

	Before USG Application		After USG Application		
Variable	Median	25–75th%	Median	25–75th%	*p*
PSV	52	40–65	47	42–63	0.807
EDV	14	11–19	14	11–16	0.143
RI	0.72	0.64–0.77	0.73	0.68–0.78	0.065

**Table 5:** Doppler ultrasonographic measurements of the patients when grouped according to the final pain scores. All measurements are given in medians, and 25th and 75th percentiles, as they were not normally distributed. Abbreviations: NPASS: Neonatal Pain, Agitation and Sedation Scale, USG: ultrasonography, PSV: peak systolic velocity, EDV: end-diastolic velocity, RI: resistive index.

	**NPASS ≤ 4 after USG (n: 15)**			**NPASS > 4 after USG (n: 24)**		
	**Before**	**After**	***p***	**Before**	**After**	***p***
PSV	65 (36–70)	55 (42–80)	0.232	52 (42–57)	46 (37–58)	0.586
EDV	16 (14–19)	15 (13–21)	0.819	13 (9–19)	13 (10–15)	0.022
RI	0.70 (0.64–0.73)	0.73 (0.68–0.76)	0.61	0.74 (0.65–0.79)	0.75 (0.67–0.79)	0.458
	**NPASS ≤ 7 after USG (n: 31)**			**NPASS > 7 after USG (n: 8)**		
	**Before**	**After**	***p***	**After**	**Before**	***p***
PSV	52 (40–66)	46 (42–67)	0.717	54 (2)	51 (35–53)	0.673
EDV	14 (11–18)	14 (11–15)	0.631	20 (16)	15 (9–20)	0.025
RI	0.72 (0.69–0.78)	0.74 (0.68–0.78)	0.410	0.63 (0.23)	0.71 (0.61–0.78)	0.036

## Data Availability

The data presented in this study are available on request from the corresponding author.
